# Tanzanian men’s engagement in household chores is associated with improved antenatal care seeking and maternal health

**DOI:** 10.1186/s12884-021-04147-z

**Published:** 2021-09-30

**Authors:** Emily Chahalis, Josie McGhie, Generose Mulokozi, Shannon Barham, Carter Chappell, Charisse Schenk, Mary Linehan, Scott Torres, Kirk A. Dearden, Josh H. West, P. Cougar Hall, Benjamin T. Crookston

**Affiliations:** 1 Brigham Young University, Provo, UT USA; 2 IMA World Health, Washington D.C, USA; 3 RTI International, Research Triangle Park, NC USA

**Keywords:** Antenatal care, Pregnancy, Male involvement, Diet, Workload, Male engagement

## Abstract

**Background:**

Male support for partners’ antenatal care (ANC) has the potential to improve women’s care-seeking and maternal health outcomes. This study describes factors that are associated with men’s involvement in household tasks and explores the relationship between men’s help with tasks and women’s ANC-seeking, diet and workload during pregnancy as well as other health behaviors.

**Methods:**

This study was conducted in five Lake Zone regions of Tanzania. Cross-sectional surveys were carried out among approximately 10,000 households that had children under the age of 2 years. Surveys were administered to mothers of children less than 2 years and where available, their male partners. Data were collected between December 2015 and May 2020, in conjunction with a large-scale campaign aimed at reducing childhood stunting by changing the behavior of mothers, caregivers, and decision makers. Data analysis included bivariate analysis and logistic regression modeling.

**Results:**

Men’s engagement in household activities was significantly associated with living in an urban setting, being younger, having at least some formal schooling, early verbal interactions with their children, and male involvement in healthcare decisions. Additionally, mothers of male partners that were engaged in household activities were significantly older and more likely to have at least some secondary school education. Relative to households where men only infrequently helped out with chores or not at all, women from households where men frequently helped were significantly more likely to have taken iron tablets during pregnancy, report having eaten more than usual, lessening their household workload during their most recent pregnancy, and more likely to have played with their child in the week prior to the survey.

**Conclusion:**

Male’s participation in household tasks is associated with a general improvement in mother’s ANC behaviors. Implicit in these findings is that general primary education for both men and women has health benefits that transcend socioeconomic class and that future interventions aimed to engage males in household tasks may target older males with less education living in rural areas.

## Introduction

In resource-poor settings, maternal health challenges such as inadequate antenatal care (ANC) persist where adequate care is lacking or unavailable [1]. One-fourth of maternal deaths occur during pregnancy due to hypertension and antepartum hemorrhaging, which has been connected to insufficient ANC [2]. Unsatisfactory access to and quality of ANC contributes significantly to these avoidable maternal deaths. A number of factors contribute to a lack of access to ANC in low-income countries, including socioeconomic status, environmental factors, education levels of mother and partner, and employment status [3]. A systematic review conducted in 2019 [3] showed that when both the woman and her partner have higher levels of education, including knowledge of the risks associated with unattended childbirth, women are more likely to attend antenatal services starting in the first trimester. Increased knowledge of ANC by both partners is predictive of increased use of antenatal services and improved maternal health outcomes. Additionally, women with previous negative experiences with physicians are less likely to participate in ANC. Due to this, women often refrain from attending recommended ANC appointments and often discourage other women from attending ANC appointments as well [4].

In many cultures, traditional male roles discourage male involvement in maternal health despite recent changes in some female roles [5, 6]. For example, in Tanzania, as women become more active in providing for their families financially, their male partners are not increasing their involvement in child care and household tasks [7]. This maintains the social norm of the mother being primarily responsible for all domestic chores in addition to her work outside the home [8]. Although men and women in this region recognize that men are capable of doing traditionally female chores, societal norms continue to dictate that men should not participate in domestic tasks [9]. This leaves some women of child bearing age exhausted and overwhelmed [10].

Whereas traditional norms often put men in control of things like family finances, male involvement in ANC could promote rearranging financial priorities for safer pregnancies due to improved knowledge of the dangers and risks of childbirth [11]. The term male involvement does not have a single definition; rather, it is a subjective term that may refer to a host of behaviors, including active participation in ANC, providing financial support for expenses related to pregnancy and childbirth, and involving the female partner in decisions regarding pre- and postnatal care [12, 13]. Male involvement is low in Tanzania [14, 15], and Tanzanian men generally do not help with tasks that are traditionally carried out by women, including cooking, cleaning, and childcare [5]. Still, recent studies suggest that males are receptive to and benefit from interventions that encourage more male involvement [5, 16].

Recent research suggests that when male partners become more involved, maternal health outcomes and women’s access to ANC may improve [17]. For example, males who are aware of the possible dangers of pregnancy and childbirth are motivated to attend ANC appointments with their partners leading to better maternal care [14, 18]. When Tanzanian men were involved in ANC, maternal and child nutrition improved [19], women’s understanding of hazards in pregnancy increased [20], and maternal depression decreased [13]. Hence, although the situational perception of men and women’s roles may initially discourage male involvement, this perception may be altered to promote higher levels of male involvement, yielding a variety of positive effects on maternal and child health [8, 11, 13, 14, 18–20].

Barriers to male involvement still remain. For example, it was recently reported that only 24% of Tanzanian men were comfortable discussing pregnancy concerns with their partner [15], and an even smaller percent (8%) of Tanzanian men were invited by their spouse to accompany them to family planning clinics [21]. Further, while Tanzanian men reported feeling that ANC was important for their wives, they did not feel motivated to participate themselves [16]. This reluctance was attributed to gender roles, undesirable health facilities, and fear [22]. However, Tanzanian men were receptive to education about important maternal health topics such as breastfeeding and reported a desire for more education during the postpartum period [5]. Additionally, some rural Tanzanian communities welcomed male involvement in previously women-only maternal health clinics [23]. Increased male involvement also had positive effects on community dynamics [23].

The purpose of the current study was to use data from the Addressing Stunting in Tanzania Early (ASTUTE) program to describe factors associated with male involvement and to explore the relationship between male involvement and maternal health and ANC outcomes as there is currently a gap in knowledge and literature surrounding these associations.

## Methods

### Description of the ASTUTE program

From 2015 to 2020, IMA World Health implemented the ASTUTE program, which was a comprehensive, regional intervention that included capacity building of nutrition officers, officers from other nutrition sensitive sectors and community-based implementors such as Community Health Workers (CHWs) and volunteers from Civil Society Organizations. Its purpose was to strengthen coordination with government nutrition sectors, and mass media and interpersonal communications (IPC) components focused on improving children’s nutrition and development indicators before a child reaches the age of 2 in order to decrease stunting. IMA World Health, in close collaboration with the Tanzanian Local Government Authorities and Ministry of Health, implemented this program with funding from UKaid and the Department for International Development (DFID). Key themes of the program included promotion of improved maternal health and nutrition during pregnancy; male engagement; exclusive breastfeeding for children 0-6 months; complementary feeding for children 6-24 months; early child development; water, sanitation, and hygiene practices; and diarrhea treatment.

The ASTUTE program was implemented in five regions of the Lake Zone in Tanzania (Geita, Kagera, Kigoma, Mwanza, and Shinyanga) with a collective population of 10.2 million and over 750,000 stunted children. These regions were selected for their documented high prevalence of stunting and anemia and poor infant and child feeding.

### Data collection and sampling

The program utilized a cross-sectional survey that was distributed to approximately 5000 households before the program was implemented and an additional 5000 households after the program was implemented. Inclusion criteria included having a child under two years (0-23 months) of age and living in the regions where the campaign took place. Respondents who did not meet these criteria were excluded from the study. Survey questions were directed to the female caregiver of the youngest child in the household, and if available, the male head of household.

Data were collected by a field team that consisted of ten supervisors and 50 enumerators. All members of the team were trained during a two-week period prior to data collection. A stratified, multi-stage random sample design was used to select participants. A pool consisting of 243 villages was chosen and used for the survey; however, participants were randomly selected during each survey round. Data were captured digitally with the use of smartphones and personal digital assistants (PDAs). IRB approval was granted by Development Media International’s (DMI) internal IRB and Tanzania’s National Institute for Medical Research. The quality of the data collected was checked by 11 supervisors, and a new interview was conducted if the quality of a previously completed interview could not be validated. The raw data were also checked for any outliers and answers that were not plausible. To maintain confidentiality, the data were only made available to the study personnel and were de-identified so that responses could not be traced back to any subjects.

### Male engagement study methodology

#### Measures

Male and men were used interchangeably and can identify a husband, father, partner, or male caregiver in the household. Female and women were used interchangeably and can identify a mother, wife, or female caregiver in the household. Chores refer to household chores such as fetching water, helping with farming, or washing clothes, etc.

Two sub-indices (access to services and consumer durables) were created and used to make up a wealth index, which is based on work by Briones [24] and was used to estimate household wealth. The first sub-index included information about safe drinking water and safe sanitation access and was used as an indicator of access to services. This index included things such as public standpipes, protected wells, water piped directly into the dwelling, and flush toilets. Pit latrines were not considered safe sanitation for this index. The second sub-index reflected ownership of eight consumer goods (radio, TV, bicycle, motorcycle, automobile, mobile phone, boat, and animal-drawn cart). These two indices were then averaged to generate an overall wealth index ranging from 0 (lowest socioeconomic status) to 1 (highest socioeconomic status).

A series of ANC and maternal health variables were also measured. Only variables relating to predictors of male engagement and household chores, and the association between male engagement and antenatal care and maternal health were explored for the study question. The criteria to choosing the existing study variables included a series of logistic regression models that showed some association between male engagement and household chores, or male engagement and antenatal care and maternal health as reported by either males or females. The primary outcome indicator for maternal health and nutrition during pregnancy was defined as the “proportion who ate more types of food during pregnancy”. The primary outcome for exclusive breastfeeding was defined as the “proportion of mothers of children 0-6 months who are currently breastfeeding and report they haven’t given the child any other food/liquids”. The primary outcome for complementary feeding was defined as the “proportion of children getting minimum meal frequency”.

#### Statistical methods and analysis

Data were cleaned and analyzed using STATA version 16 (College Station, TX). *P*-values were used to assess the strength of associations between study variables and were obtained using Likelihood Ratio Tests. Sample proportions, odds ratios (ORs), and 95% confidence intervals were used for estimating the strength of each association. All logistic regression models controlled for household wealth using the created wealth index, male age, and male education.

## Results

### Sample demographics

Responses from 8308 study participants were analyzed from the survey. The majority of men (47.5%) and women (44.7%) surveyed were between ages 26-35. The majority of both men (64.2%) and women (56.8%) reported having completed primary school (see Table 1). The majority of men reported being self-employed as their household’s source of income (86.9%), and the majority of females reported farming and fishing as their occupation (75.1%). The majority of households’ wealth indices fell between 0.26-0.75 (65.8%).

**Table 1:** Household Characteristics of Male and Female Study Participants in Tanzania

Male Characteristics	Frequency, n	Percentage, %
**Age**		
< 25	551	13.1
26-35	2004	47.5
36-45	1185	28.1
46+	477	11.3
**Education**		
Less than primary school	744	17.5
Completed primary school	2732	64.2
Some secondary or more	779	18.3
**Source of Income**		
Pension, NGO aid, or family member	70	1.7
Outside employment	465	11.0
Self-employed	3672	86.9
No source of income	19	0.5
**Female Characteristics**		
**Age**		
< 25	1649	40.3
26-35	1827	44.7
36-45	555	13.6
46+	60	1.5
**Education**		
Less than primary school	1331	31.6
Completed primary school	2394	56.8
Some secondary or more	491	11.7
**Occupation**		
Farming/Fishing	3167	75.1
Employed by govt or NGO	93	2.2
Self-employed	435	10.3
Paid/unpaid family helper	5	0.1
Unemployed/student	516	12.2
**Wealth Index**^**a**^		
< .25	3356	33.6
.26 - .75	6572	65.8
.76+	68	0.7

^a^wealth index based on female responses

### Predictors of male engagement

#### Reported by women

As male age increased, males were significantly less likely to help with chores (OR = 0.99) (see Table 2). Males were more likely to participate in chores when either men or women had completed some secondary education, or when women completed primary education (OR = 1.64, 1.35, 1.21). When males made healthcare decisions independently or with women, the male’s involvement in chores increased (OR = 1.33, 1.29). Males were less likely to help with chores as the number of children under 5 increased (OR = 0.88). Males were also less likely to help with chores as the age of the child’s first verbal engagement with the male increased (OR = 0.97). Women also reported that males were less likely to assist with chores when living in rural areas (OR = 0.77).

**Table 2:** Predictors of Male Engagement^a^ in the Household as Reported by Female Study Participants in Tanzania

Variables	Odds Ratio (95% CI)	***p***-value
Wealth	0.84 (0.58-1.20)	0.334
Male completed some primary education	1.06 (0.89-1.25)	0.521
Male completed some secondary education	1.35 (1.09-1.68)	0.007
Female completed some primary education	1.21 (1.05-1.40)	0.010
Female completed some secondary education	1.64 (1.29-2.08)	0.000
Father’s age	0.99 (0.99-1.00)	0.035
Mother’s age	0.99 (0.98-1.00)	0.176
Number of children under the age of 5 in the household	0.88 (0.80-.97)	0.012
Sex of child is male	0.93 (0.82-1.06)	0.256
Child’s age when father started talking to child	0.97 (0.95-0.99)	0.009
Decisions about healthcare: Male only	1.29 (1.08-1.54)	0.006
Decisions about healthcare: Both the female and male	1.33 (1.11-1.60)	0.002
Living in a rural setting	0.77 (0.62-0.95)	0.013

^a^Male engagement was defined as the male frequently helping with household chores

Controlled with male education, male age, and wealth

#### Reported by men

Males were more likely to help with chores as the women’s age increased (*p*-value = .026) (see Table 3). Males were also more likely to help with chores if they completed either primary or secondary education (OR: 1.23, *p*-value = 1.610). Additionally, when the females completed some secondary education, males were more likely to help with chores (OR: 1.27, *p*-value = .042). A woman’s completion of primary education did not significantly increase the likelihood that the male helped with chores (*p*-value = .411). However, males who made healthcare decisions, either independently or with the women, were 1.29 and 1.33 times more likely to report that they did household chores as compared to males who reported that the female made healthcare decisions independently (CI = 1.11-1.59, *p*-value = .002).

**Table 3:** Predictors of Male Engagement^a^ in the Household as Reported by Male Study Participants in Tanzania

Variables	Odds Ratio (95% CI)	***p***-value
Wealth	0.70 (.49-1.00)	0.053
Male completed some primary education	1.23 (1.04-1.45)	0.014
Male completed some secondary education	1.61 (1.30-1.99)	< 0.001
Female completed some primary education	1.06 (.92-1.22)	0.413
Female completed some secondary education	1.27 (1.01-1.61)	0.042
Father’s age	1.00 (.99-1.00)	0.319
Mother’s age	1.02 (1.00-1.03)	0.026
Number of children under the age of 5 in the household	0.96 (.88-1.06)	0.411
Sex of child is male	0.92 (.81-1.04)	0.184
Child’s age when father started talking to child	1.01 (.99-1.04)	0.244
Decisions about healthcare: Male only	1.33 (1.11-1.59)	0.002
Decisions about healthcare: Both the female and male	1.14 (.95-1.37)	0.152
Living in a rural setting	0.92 (.75-1.12)	0.400

^a^Male engagement was defined as the male frequently helping with household chores

Controlled with male education, male age, and wealth

### Association between male engagement and antenatal care and maternal health

#### Reported by women

Women who reported that males helped with chores were 1.90 times more likely to have taken iron tablets to prepare for pregnancy (*p*-value = 0.006), and to have eaten more food than usual and reduced their household workload during their pregnancy (*p*-value = 0.000) (see Table 4). At the time of the survey, women whose male partners assisted with chores were also 1.37 times more likely to report having spent time playing with the child in the past week.

**Table 4:** Association Between Male Engagement^a^ and ANC and MH as Reported by Female Study Participants in Tanzania

Variables	Frequency, n	Percentage, %	Odds Ratio (95% CI)	***p***-value
Female ate more food during pregnancy	589/9346	6.3	1.67 (1.34-2.00)	< 0.001
Female worked less in the household during pregnancy	3184/9355	34.0	2.25 (1.96-2.58)	< 0.001
Disagree that during pregnancy, women should continue to carry out the same chores as normal	1642/9357	17.6	1.04 (.91-1.18)	0.582
Female saw someone for antenatal care	2751/9344	29.4	1.08 (.93-1.24)	0.307
Before becoming pregnant, female took iron tablets	244/4653	5.2	1.90 (1.20-3.00)	0.006
Exclusively breastfed child under 6 months	1010/3269	30.9	1.05 (.83-1.34)	0.665
Introduced complementary foods to child between the age of 6 and 8 months	413/1231	33.6	1.18 (.79-1.74)	0.423
Child met guidelines for dietary diversity (4 or more food groups out of 6)	758/3083	24.6	0.94 (.79-1.12)	0.498
Spent time playing with child in the last week	3296/9347	35.3	1.37 (1.19-1.59)	< 0.001

^a^Male engagement was defined as the male frequently helping with household chores

Controlled with male education, male age, and wealth

#### Reported by men

In households where males reported helping with chores, females were 1.62 times more likely to report that they ate more than usual at some point during their pregnancy (see Table 5). Males were also more likely to report that the woman lessened her household workload during her most recent pregnancy (*p* = <.001) and more likely to have spent time playing with her child in the past week (*p*-value = .013).

**Table 5:** Association Between Male Engagement^a^ and ANC and MH as Reported by Male Study Participants in Tanzania

Variables	Frequency, n	Percentage, %	Odds Ratio (95% CI)	***p***-value
Female ate more food during pregnancy	335/3959	8.5	1.62 (1.32-1.98)	< 0.001
Female worked less in the household during pregnancy	1721/3964	43.4	1.71 (1.49-1.97)	0.000
Disagree that during pregnancy, women should continue to carry out the same chores as normal	916/3975	23.0	1.04 (.92-1.19)	0.528
Female saw someone for antenatal care	1715/3965	43.3	1.09 (.95-1.26)	0.229
Before becoming pregnant, female took iron tablets	59/901	6.6	1.42 (.92-2.20)	0.117
Exclusively breastfed child under 6 months	610/1476	41.3	1.03 (.83-1.29)	0.771
Introduced complementary foods to child between the age of 6 and 8 months	222/520	42.7	0.83 (.55-1.24)	0.355
Child met guidelines for dietary diversity (4 or more food groups out of 6	564/2000	28.2	0.91 (.76-1.09)	0.287
Spent time playing with child in the last week	1885/4217	44.7	1.20 (1.04-1.37)	0.013

^a^Male engagement was defined as the male frequently helping with household chores

Controlled with male education, male age, and wealth

## Discussion

The purpose of this study was to identify factors associated with male involvement in traditional household female tasks and the extent to which involvement was associated with ANC and maternal health. A positive association was found between male involvement at home with chores and ANC and maternal health.

The current study identifies several key predictors of male involvement. According to responses by females, results demonstrate that males were more likely to be involved in helping with chores when they had completed some secondary education and made healthcare decisions independently or with the female in the household. From the males’ perspective, males were more likely to help with chores as the female’s age increased, males completed either primary or secondary education, and males made healthcare decisions, either independently or with the female.

Findings of the current study are consistent with studies in other countries. Researchers found that females had the greatest burden of chores when both the male and the female had lower levels of education, while higher education levels are associated with a more equitable share of chores between males and females [25]. A study conducted in India demonstrated that demographic variables, such as age and education, can directly and indirectly affect joint decision-making toward healthcare and male engagement within the home [26]. Results from both studies are consistent with findings presented here that as age, education, and making joint health decisions increase, males help more with household chores.

The current study found that when males and females reported that the male assisted with chores more frequently, females were more likely to report eating more during pregnancy, lessening their workload while pregnant, and spending more time playing with their child in the past week. Studies have shown that when females play with their child it improves early development [27] and that heavy workloads during pregnancy lead to lower birth weights or smaller fetal size [28]. These findings lend support to the idea that when males assist with chores, females are less overworked and benefit from improved maternal health. These health benefits to the mother potentially translate into improved health for the child.

The findings of this study also suggest a positive association between male involvement in the home and the likelihood of a woman taking iron tablets in preparation for and during pregnancy. Iron is critical to a healthy pregnancy and assists in preventing anemia, preterm delivery, and low birth weight [29–31]. Iron supplementation during pregnancy is especially important in areas such as Tanzania where iron deficiency is endemic [30]. Previous studies suggest that iron supplementation rates in Tanzania are low [32], so the potential connection between male engagement in the home and taking iron tablets for pregnancy merits further research and verification.

This study introduced new knowledge that while recent emphasis has been placed on educating women, when men are educated above primary school there is a positive effect on ANC and maternal health outcomes. Educated males are more likely to be involved with healthcare decision making than those with lower levels of education. Furthermore, when men and women are both involved with healthcare decision making ANC and maternal health improve. This finding also shows that both men and women need to be informed about effective healthcare practices to improve these decisions.

Also, as men participate in household chores, several benefits in ANC compliance and participation were noted. Male involvement had a positive impact on women’s ability to eat better, rest and take their iron tablets. These actions will help women have better birth outcomes. Additionally, an increase in women playing with their young child was identified, which can improve their social and academic skills. Cultural norms are difficult to change, but as these desirable ANC and maternal health outcomes are seen, progress will be made.

These findings suggest that both healthcare providers and policymakers could be involved in improving ANC. Healthcare providers can encourage and even request male involvement in ANC visits, education and decision making. Policymakers’ role could include education initiatives teaching that male involvement in household chores improves ANC and maternal outcomes, policies that support education beyond primary school for males and females, and affordable and accessible ANC including appointments and supplemental tablets.

## Limitations

The results of this study should be interpreted in the context of several limitations. This study was conducted in five Lake Zone regions of Tanzania and the results cannot be applied generally to all of Tanzania or the African continent. However, the sample was selected carefully and represents a broad demographic from the region. Another limitation is the data used came from a large-scale intervention designed to improve broad maternal and child health outcomes. As a result, the number of indicators specifically investigating male involvement are limited. Despite these limitations, this study contributes to the current research regarding the effects of male attitudes, knowledge, and behaviors on ANC.

## Conclusion

Male involvement in household chores is significantly associated with key factors that contribute to improved maternal and young child health and development. Our findings are suggestive of a positive relationship between male involvement in the home and maternal health, which in turn benefits the health and development of the child. All of these male involvement predictors remained true even when accounting for socioeconomic factors. These findings are encouraging because they suggest that, at any income level, younger, more educated men in urban areas may be early adopters of behaviors that contradict traditional gender norms and benefit women and children. Future interventions aimed at improving maternal and child health may benefit from considering how males can be targeted and influenced to improve the health and wellbeing of the entire household.

## Data Availability

The datasets analyzed during the current study are available from the corresponding author on reasonable request.
